# An Exploratory Assessment of Geographic Variation and Disease Clustering of Multimorbidity Among Older Adults in Odisha, India

**DOI:** 10.7759/cureus.110436

**Published:** 2026-06-08

**Authors:** Prashansa Das, Manas Ranjan Behera, Aurolipy Das, Deepanjali Behera, Junaid Khan

**Affiliations:** 1 School of Public Health, Kalinga Institute of Industrial Technology (KIIT) Deemed to be University, Bhubaneswar, IND; 2 Faculty of Management Sciences, Institute of Business and Computer Studies (IBCS), Siksha 'O' Anusandhan Deemed to be University, Bhubaneswar, IND; 3 Hospital Administration, Faculty of Management Sciences, Institute of Business and Computer Studies (IBCS), Siksha 'O' Anusandhan Deemed to be University, Bhubaneswar, IND; 4 Biostatistics, Vivekananda College, Kolkata, IND

**Keywords:** disease clustering, geriatric care, multi-morbid, older adults, spatial analysis

## Abstract

Background: Multimorbidity among older adults is a significant public health issue in India, yet its regional distribution within states remains poorly understood. Most existing research focuses on overall prevalence or individual risk factors, with less emphasis on district-level differences, spatial heterogeneity, and disease clustering. This study aimed to assess the prevalence, geographic variation, and disease co-occurrence patterns of multimorbidity among older adults in six selected districts of Odisha, India.

Materials and methods: A community-based cross-sectional study involving 1,072 adults aged 60 and above was carried out across six districts of Odisha: Khordha, Cuttack, Balasore, Sundargarh, Ganjam, and Sambalpur. Multimorbidity was defined as the presence of two or more self-reported physician-diagnosed chronic conditions in the same participant. The prevalence was estimated for each district, and multivariable binary logistic regression was used to analyze factors linked to multimorbidity. Spatial heterogeneity was evaluated using Global Moran’s I, and district-specific disease-pair clustering was examined using Jaccard similarity matrices.

Results: A total of 677 participants had multimorbidity, representing a prevalence of 63.2%. The burden varied widely across districts, from 54.2% in Khordha to 74.8% in Cuttack. Sundargarh also exhibited a high prevalence of 73.6%. Spatial analysis revealed significant heterogeneity in the distribution of multimorbidity, with a Global Moran’s I of -0.5082, suggesting that high-burden areas were unevenly distributed across the selected districts. Cuttack and Sundargarh stood out as key high-burden regional outliers. In the adjusted analysis, factors such as district of residence, multiple medication use, and living arrangements were linked to multimorbidity. Older adults living only with children had higher odds than those living with both a spouse and children. Jaccard similarity analysis identified district-specific morbidity patterns, including stronger respiratory-musculoskeletal clustering in Khordha and a consistent hypertension-diabetes clustering across districts.

Conclusion: Multimorbidity among older adults in Odisha shows clear regional variations and specific disease groupings. These results imply that a uniform approach may not be sufficient for managing geriatric multimorbidity across different district contexts. Instead, tailored district-level integrated care, regular medication reviews, and locally adapted multimorbidity management strategies could lead to more sustainable elderly care in Odisha.

## Introduction

Population aging represents one of the key demographic and public health shifts of the 21st century [1]. The rise in life expectancy has brought significant benefits to society, yet it has also led to a greater burden of chronic illnesses, functional decline, and long-term healthcare requirements in older adults [2,3]. The World Health Organization defines healthy aging as the process of developing and maintaining functional abilities that enable well-being in older age. This perspective emphasizes not only disease control but also functional capacity, independence, dignity, and quality of life among older adults. In this context, multimorbidity becomes an important concern because the coexistence of multiple chronic conditions can reduce functional ability, increase treatment complexity, and create a greater need for integrated and person-centered care [4]. A key outcome of this process is the emergence of multimorbidity, defined as having at least two chronic conditions simultaneously [5,6]. Generally, around one-third of adults worldwide experience multimorbidity, with an even higher prevalence among older adults [7]. Furthermore, in developing countries, the issue is worsened by health systems concentrating on specific diseases, limiting their ability to care for the aging population [8]. India is at a critical stage in its epidemiologic transition. It has the world's largest aging population, with 149 million people in 2022, projected to grow to 347 million by 2050. Meanwhile, the burden of chronic diseases is rapidly rising among the elderly population. It is projected that the proportion of people over 60 in India will almost double, reaching 19.5% by 2050 [9]. Research from the Longitudinal Ageing Study in India (LASI) shows that multimorbidity is common in India today, with higher risks among people who live in cities and those who are single [10]. Odisha provides an excellent context for studying geriatric multimorbidity [11]. The state comprises a mix of predominantly rural communities and areas undergoing industrialization and urbanization [12]. Odisha includes predominantly rural populations along with urbanizing and industrial regions, making it important to examine whether multimorbidity patterns vary across different geographic and service contexts [13]. Socioeconomic differences are among the determinants of health that have been extensively discussed in previous studies, but much remains to be studied regarding how specific geographic settings affect geriatric multimorbidity [14]. Similarly, the ways in which geography can affect health, such as increasing medication burden and weakening traditional family or spousal support systems, are still not well understood [15,16]. There is a clear need for region-specific evidence that goes beyond reporting overall prevalence and examines geographic variation and disease co-occurrence patterns among older adults. Understanding how multimorbidity burden varies across districts is important for shifting from a fragmented, reactive healthcare system to a proactive, person-centered approach, as promoted by the WHO ICOPE (World Health Organization Integrated Care for Older People) framework. In this context, this study aimed to assess the prevalence, geographic variation, and disease co-occurrence patterns of multimorbidity among older adults in six selected districts of Odisha, India, and to examine selected sociodemographic and clinical factors associated with multimorbidity.

## Materials and methods

A community-based, cross-sectional analytical study was conducted among older adults in Odisha, India. The geographic unit of analysis for this study was the selected district. The study was carried out in six selected districts: Khordha, Cuttack, Balasore, Sundargarh, Ganjam, and Sambalpur. Within these districts, urban wards with a higher concentration of older adults were selected for data collection. Bhubaneswar and Berhampur were considered urban locations within Khordha and Ganjam districts, respectively. The districts were selected to capture broad regional variation within Odisha, including administrative, coastal-commercial, industrial, southern, and western contexts. Khordha, represented by Bhubaneswar, reflects the state’s administrative and healthcare hub; Cuttack represents a major urban and healthcare center; Sundargarh reflects an industrial setting; Balasore represents a coastal-commercial region; Ganjam, represented by Berhampur, reflects southern urban-market dynamics; and Sambalpur represents western Odisha. This regional diversity enabled the study to explore how the burden and patterns of multimorbidity vary across different geographic and service contexts. The findings should therefore be interpreted as observations from selected districts rather than as a fully representative spatial assessment of all districts of Odisha.

**Figure 1:**
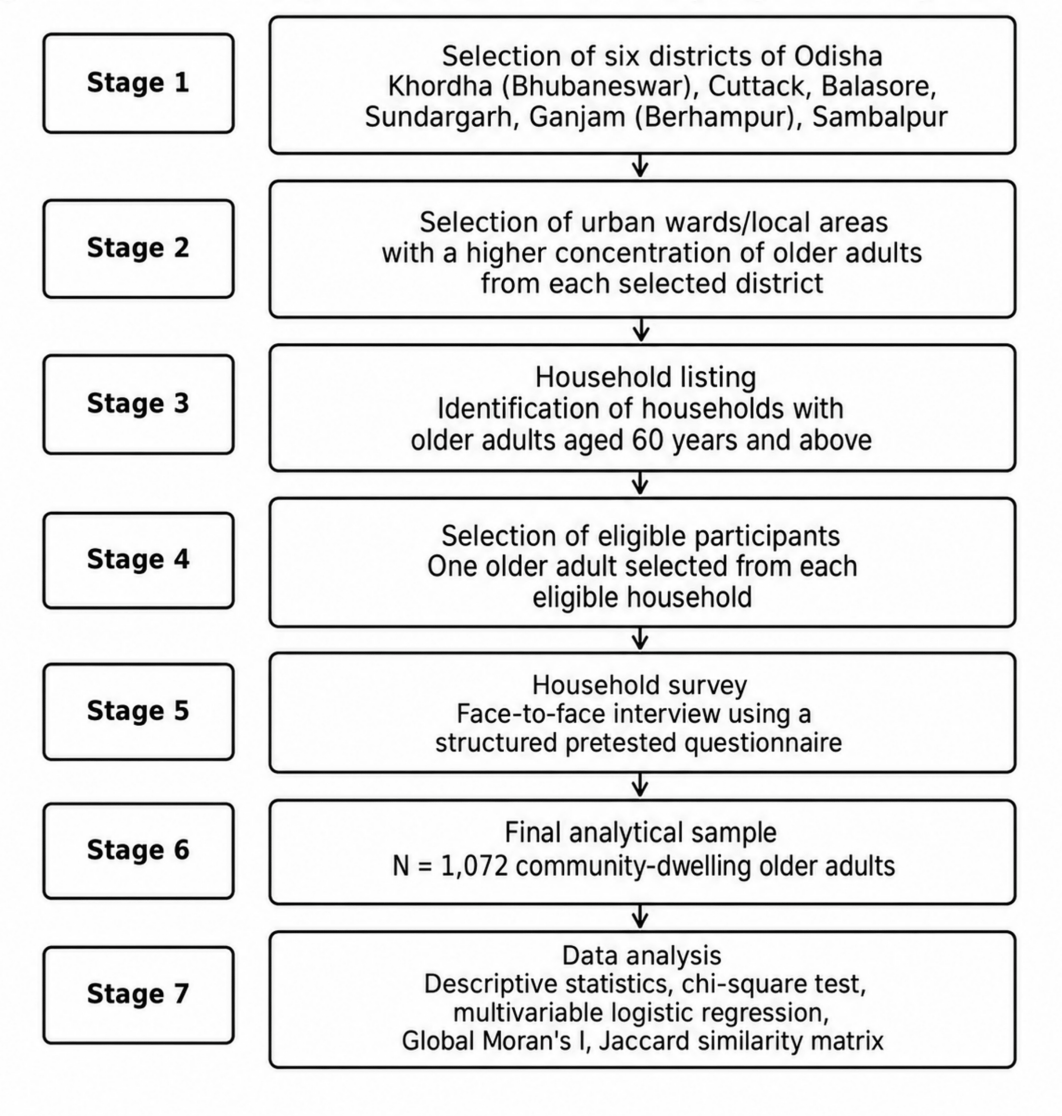
Schematic presentation of the multistage sampling and analytical flow. The figure shows the study flow from selection of six districts and urban wards/local areas to household listing, participant recruitment, final sample inclusion, and statistical, spatial, and disease-clustering analyses.

The study included 1,072 community-dwelling older adults from six selected districts of Odisha: Khordha, Cuttack, Balasore, Sundargarh, Ganjam, and Sambalpur. Within these districts, urban wards with a higher concentration of older adults were selected for data collection. A multistage sampling method was followed. First, the six districts were selected to capture broad regional variation across Odisha. Next, municipal wards or local administrative areas were selected within each district. Household listing was then conducted in the selected wards to identify households with at least one eligible older adult aged 60 years or above. Eligible households were approached systematically, and one older adult was selected from each household. If more than one eligible older adult was present in a household, one participant was selected randomly (Figure 1).

The sample size was estimated using the single population proportion formula:

$n = \frac{Z^{2}\times p\times (1 &minus; p)}{E^{2}}$

where Z is the standard normal value at the 95% confidence level, p is the expected prevalence, and E is the margin of error. Since the true prevalence of geriatric multimorbidity in the study setting was unknown, a conservative estimate of 50% was used. Taking Z = 1.96, p = 0.50, and E = 0.03, the required sample size was approximately 1,067. After applying the finite population correction, the adjusted sample size was approximately 1,066. The final analytical sample comprised 1,072 older adults, exceeding the minimum required sample size. Participants were eligible if they were aged 60 years or older, residents of the selected area for at least 6 months prior to data collection, able to understand Odia, Hindi, or English, and willing to provide informed consent. Bedridden, terminally ill, severely cognitively impaired individuals and those who refused to participate were excluded.

Data were collected through face-to-face interviews using a structured and pretested questionnaire administered by trained interviewers. The questionnaire was adapted from the LASI Wave 1 survey instrument, particularly the sections related to sociodemographic profile, chronic disease history, living arrangements, healthcare use, and medication use. The tool was modified according to the study objectives and local context, reviewed by subject experts, and pretested before final data collection. The main dependent variable was multimorbidity, defined as the presence of two or more self-reported physician-diagnosed chronic conditions in the same participant. Chronic conditions were assessed by asking participants whether a doctor or health professional had ever diagnosed them with the condition. The conditions included in the analysis were hypertension, diabetes mellitus, respiratory conditions, and musculoskeletal conditions. Hypertension and diabetes mellitus were based on self-reported prior physician diagnosis. Respiratory conditions included self-reported physician-diagnosed chronic respiratory illnesses such as asthma, chronic bronchitis, or chronic obstructive pulmonary disease. Musculoskeletal conditions included self-reported physician-diagnosed arthritis, rheumatism, osteoporosis, or chronic joint-related disorders. These conditions were not clinically measured during the survey and were not verified through medical records. Based on the morbidity count, participants with two or more conditions were classified as having multimorbidity, while those with fewer than two conditions were classified as not having multimorbidity. The independent variables included age, gender, education, household monthly income, living arrangement, and district of residence. Medication burden was assessed by counting the number of prescribed medications taken daily. 

Data analysis was conducted using IBM SPSS Statistics for Windows, Version 26 (Released 2018; IBM Corp., Armonk, New York) and GeoDa version 1.20 (GeoDa Center, Chicago, Illinois) for spatial analyses [17]. Descriptive statistics summarized the demographic characteristics of the study population, with categorical variables expressed as frequencies and percentages. The association between multimorbidity and variables such as age, gender, education, income, and district of residence was examined using Pearson’s chi-squared test. Multivariable binary logistic regression was performed using the enter method to identify independent predictors of multimorbidity. Results are presented as adjusted odds ratios (aORs) with 95% confidence intervals (CIs).

Spatial analysis was performed using GeoDa version 1.20. Global Moran’s I assessed spatial autocorrelation in multimorbidity prevalence across the study sites. A negative Moran’s I signified spatial heterogeneity, indicating that high-burden regions were close to or surrounded by lower-burden regions. Clustering of disease pairs was examined using Jaccard similarity matrices, where specific chronic conditions were represented as binary variables. The Jaccard index measured the degree of overlap between condition pairs, representing the co-occurrence of two conditions in the same individuals, where higher values signified stronger co-occurrence. The analysis focused on common conditions such as hypertension, diabetes mellitus, respiratory diseases, and musculoskeletal conditions [18].

Ethical approval for this study was obtained from the Institutional Ethics Committee of Kalinga Institute of Medical Sciences, Kalinga Institute of Industrial Technology (KIIT) Deemed to be University, Bhubaneswar, Odisha, India. The approval reference number was KIIT/KIMS/IEC/2006/2025, dated February 28, 2025. Data were collected from March 2025 to December 2025. Before enrollment, participants were informed about the purpose of the study, the voluntary nature of participation, the confidentiality of responses, and their right to withdraw at any stage. Written informed consent was obtained from all participants before data collection. Participant anonymity was maintained by assigning codes, and no personally identifiable information was used during analysis or reporting.

## Results

The study analyzed a total of 1,072 older adults. Among them, 677 participants had multimorbidity, representing a prevalence of 63.2%. Details of the sociodemographic and clinical characteristics are provided in Table 1. The majority were from the younger-old age groups, with 33.9% aged 60-64 years and 32.6% aged 65-69 years. Women comprised a larger proportion of the sample than men (57.4% versus 41.4%). Nearly half (49.7%) of the participants had completed at least secondary education or higher, and 72.4% were currently married. Economically, the middle- (35.7%) and upper-middle-income (30.6%) levels were the most common. Multimorbidity was significantly associated with various variables in bivariate analysis using Pearson’s chi-square test. District of residence was a particularly important factor (p < 0.001), highlighting notable regional differences in health status across Odisha. Clinical complexity was also evident in medication burden, with 45.9% of participants taking more than one medication daily (p < 0.001). Additionally, significant associations were found with social determinants of health, including educational level (p = 0.022), income level (p = 0.026), and living situation (p = 0.035). Overall, these findings suggest that socioeconomic factors, living arrangements, district of residence, and medication burden were associated with multimorbidity in bivariate analysis.

**Table 1:** Sociodemographic and clinical characteristics of the study population and their association with multimorbidity status (N = 1,072). Multimorbidity was defined as the presence of two or more self-reported physician-diagnosed chronic conditions in the same participant. Chronic conditions were not clinically measured during the survey or verified through medical records. Values are presented as frequency (n) and percentage (%). Percentages in the multimorbidity-status columns are row percentages. P-values were calculated using Pearson’s chi-square test. A p-value < 0.05 was considered statistically significant.

Variable	Category	Total N (%)	Without multimorbidity n (%)	With multimorbidity n (%)	p-value
Age group	60–64 years	364 (33.9)	117 (32.1)	247 (67.9)	0.002
	65–69 years	350 (32.6)	137 (39.1)	213 (60.9)	
	70–74 years	224 (20.9)	103 (46.0)	121 (54.0)	
	75–79 years	91 (8.5)	26 (28.6)	65 (71.4)	
	80+ years	43 (4.0)	12 (27.9)	31 (72.1)	
Gender	Male	444 (41.4)	157 (35.4)	287 (64.6)	0.475
	Female	616 (57.4)	232 (37.7)	384 (62.3)	
	Other	12 (1.1)	6 (50.0)	6 (50.0)	
District	Khordha/Bhubaneswar	238 (22.2)	109 (45.8)	129 (54.2)	<0.001
	Cuttack	214 (19.9)	54 (25.2)	160 (74.8)	
	Balasore	107 (10.0)	43 (40.2)	64 (59.8)	
	Sundargarh	193 (18.0)	51 (26.4)	142 (73.6)	
	Ganjam/Berhampur	160 (14.9)	70 (43.8)	90 (56.3)	
	Sambalpur	160 (14.9)	68 (42.5)	92 (57.5)	
Education	No formal education	65 (6.1)	17 (26.2)	48 (73.8)	0.022
	Primary/middle	251 (23.4)	109 (43.4)	142 (56.6)	
	Secondary/higher secondary	533 (49.7)	179 (33.6)	354 (66.4)	
	Graduation	201 (18.7)	81 (40.3)	120 (59.7)	
	Post-graduation and above	22 (2.1)	9 (40.9)	13 (59.1)	
Marital status	Currently married	777 (72.5)	296 (38.1)	481 (61.9)	0.113
	Widowed/single	295 (27.5)	99 (33.6)	196 (66.4)	
Income level	Low income	24 (2.2)	8 (33.3)	16 (66.7)	0.026
	Lower middle	133 (12.4)	44 (33.1)	89 (66.9)	
	Middle	383 (35.7)	156 (40.7)	227 (59.3)	
	Upper middle	328 (30.6)	101 (30.8)	227 (69.2)	
	High income	204 (19.0)	86 (42.2)	118 (57.8)	
Living arrangement	Living alone	157 (14.6)	50 (31.8)	107 (68.2)	0.035
	With spouse only	94 (8.8)	34 (36.2)	60 (63.8)	
	With spouse and children	533 (49.7)	209 (39.2)	324 (60.8)	
	With children	134 (12.5)	39 (29.1)	95 (70.9)	
	With other relatives/friends	148 (13.8)	63 (42.6)	85 (57.4)	
	Institutional care	6 (0.6)	0 (0.0)	6 (100.0)	
Medication burden	Multiple medications	492 (45.9)	150 (30.5)	342 (69.5)	<0.001
	Single/no medication	580 (54.1)	245 (42.2)	335 (57.8)	

**Figure 2:**
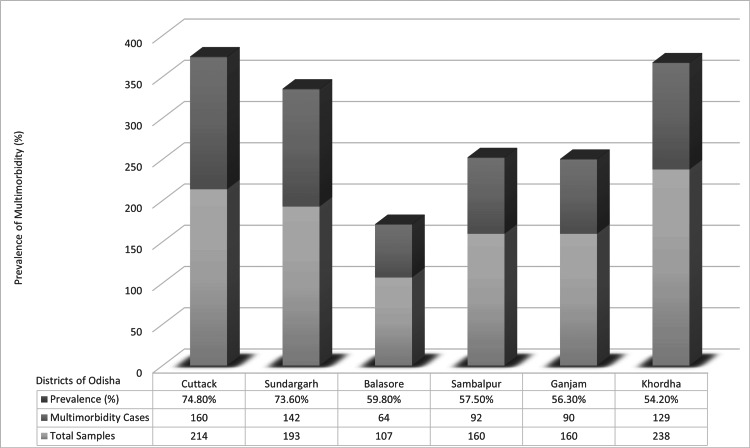
Prevalence of multimorbidity across six districts in Odisha, India (N = 1,072). Multimorbidity was defined as the presence of two or more self-reported physician-diagnosed chronic conditions. The labels on the graph indicate the percentage of respondents with multimorbidity in each district. Notably, the prevalence varies significantly across different regions (p<0.001).

The prevalence of multimorbidity varied significantly across the six selected districts, as shown in Figure 2. Cuttack had the highest prevalence at 74.8%, followed by Sundargarh at 73.6%. Balasore had a prevalence of 59.8%, while Sambalpur and Ganjam had rates of 57.5% and 56.3%, respectively. Khordha had the lowest prevalence at 54.2%. These results demonstrate notable regional differences in the burden of multimorbidity among older adults in Odisha. 

Multivariable binary logistic regression was conducted to identify factors independently linked to multimorbidity, controlling for selected sociodemographic and clinical variables (Table 2). District of residence, living arrangements, and medication load remained important predictors. Compared with Khordha, older adults from Cuttack, Sundargarh, and Balasore had significantly higher odds of multimorbidity. Those living only with children had increased odds compared with those living with both a spouse and children. Multiple medication use was significantly associated with multimorbidity, with participants taking two or more prescribed medications daily having higher odds of multimorbidity than those taking a single medication or no medication (aOR = 1.81; 95% CI: 1.32-2.47; p < 0.001). 

**Table 2:** Multivariable binary logistic regression analysis of factors associated with multimorbidity among older adults (N = 1,072). Multivariable binary logistic regression was performed with multimorbidity status as the dependent variable. The model included gender, age group, district, education, income level, living arrangement, and medication burden. Results are presented as adjusted odds ratios with 95% confidence intervals. Complete-case analysis was used; all 1,072 participants had complete data for variables included in the model. Income level was referenced to the middle-income category to improve model stability because of the small low-income group. The institutional care category was merged with joint family/others due to the small number of observations. Model fit: log-likelihood = -662.84; Nagelkerke R² = 0.105. Multicollinearity was assessed using the variance inflation factor, and all VIF values were below 10. District was included to account for geographic variation; ward-level clustering was not separately modeled. The reference category is labeled as “Ref.” A p-value under 0.05 was deemed statistically significant.

Variable	Category	Adjusted OR (aOR)	95% CI	p-value
Gender	Male (Ref)	1.00	—	—
	Female	0.80	0.61–1.06	0.117
	Other	0.56	0.16–1.96	0.365
Age group	60-64 years (Ref)	1.00	—	—
	65-69 years	0.71	0.51-0.99	0.044
	70-74 years	0.53	0.36-0.80	0.002
	75-79 years	0.92	0.52-1.64	0.785
	80+ years	1.17	0.54-2.56	0.693
District	Khordha (Ref)	1.00	—	—
	Balasore	1.83	1.05-3.21	0.034
	Ganjam	0.86	0.50-1.48	0.592
	Cuttack	2.14	1.33-3.45	0.002
	Sambalpur	1.58	0.95-2.61	0.076
	Sundargarh	2.39	1.44-3.98	<0.001
Education	No formal education (Ref)	1.00	—	—
	Primary/middle	0.74	0.36-1.55	0.431
	Secondary/higher secondary	1.16	0.57-2.36	0.680
	Graduation	0.89	0.43-1.84	0.759
	Post-graduation and above	0.84	0.28-2.52	0.756
Income level	Middle income (Ref)	1.00	—	—
	Low income	1.09	0.42-2.83	0.854
	Lower middle	1.29	0.82-2.01	0.270
	Upper middle	1.31	0.93-1.87	0.127
	High income	1.08	0.70-1.67	0.727
Living arrangement	With spouse and children (Ref)	1.00	—	—
	Living alone	1.37	0.81-2.32	0.238
	With spouse only	1.20	0.72-2.01	0.482
	With children only	1.75	1.11-2.75	0.015
	Joint family/others/institutional care	1.30	0.85-1.97	0.229
Medication burden	Single/no medication (Ref)	1.00	—	—
	Multiple medications	1.81	1.32-2.47	<0.001

**Figure 3:**
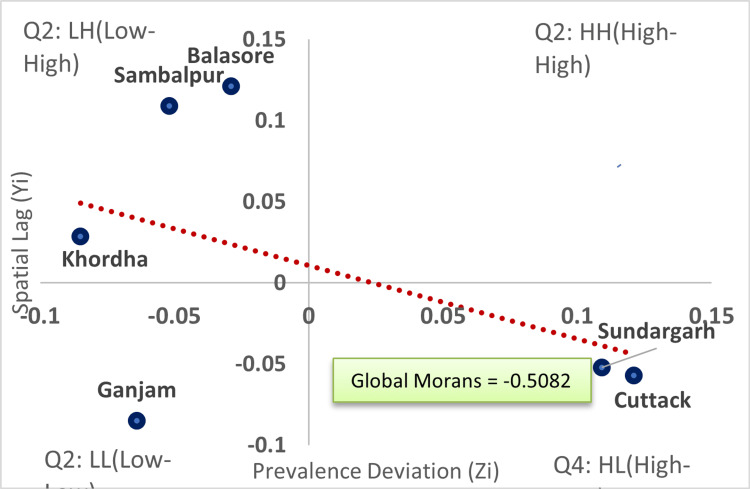
Moran scatterplot of multimorbidity prevalence across six selected districts in Odisha. The plot highlights the spatial autocorrelation of multimorbidity among the study districts (N = 1,072). The negative Moran’s I value (I = -0.5082) indicates notable spatial heterogeneity in the distribution of multimorbidity. Districts such as Cuttack and Sundargarh are positioned within the high-low quadrant, indicating that they are high-burden spatial outliers relative to the spatial lag of the selected districts.

Global Moran’s I analysis was performed to assess spatial autocorrelation in multimorbidity prevalence across the selected districts. The analysis yielded a Moran’s I value of −0.5082, with statistical significance at p < 0.05. This negative value indicates spatial heterogeneity or spatial contrast, suggesting that high-burden areas were unevenly distributed across the selected districts. The Moran scatterplot identified Cuttack and Sundargarh as high-burden regional outliers. As shown in Figure 3, these regions were located in the high-low quadrant, indicating that multimorbidity burden was concentrated in specific geographic areas rather than being evenly distributed. 

Disease-pair clustering was examined using Jaccard similarity matrices for selected districts (Table 3). In Khordha, the strongest overlap was between respiratory and musculoskeletal conditions, with a Jaccard index of 0.3158. In Sundargarh, this respiratory-musculoskeletal overlap was lower, with a Jaccard index of 0.1639. Hypertension and diabetes showed consistent clustering in both regions, with Jaccard indices of 0.1858 in Khordha and 0.1983 in Sundargarh. These findings suggest that, although metabolic disease clustering is common across regions, some disease-pair relationships may vary according to local context. 

**Table 3:** Comparative Jaccard similarity matrices showing morbidity clustering in Khordha and Sundargarh. The Jaccard similarity index (J), which ranges from 0 to 1, indicates the likelihood of two diseases co-occurring in the same individual, with higher values representing a stronger association. These matrices reveal district-specific clustering patterns for four chronic conditions: hypertension (HTN), diabetes mellitus (DM), respiratory conditions (Resp), and musculoskeletal conditions (Musculo). The data are based on a cross-sectional study of older adults in Odisha (N = 1,072).

Khordha							Sundargarh					
HTN	1	0.1858	0.2086	0.2314	1	Jaccard Index	HTN	1	0.1983	0.1707	0.1524	1
DM	0.1858	1	0.142	0.0875	0.8		DM	0.1983	1	0.0556	0.1111	0.8
Resp	0.2086	0.142	1	0.3158	0.6		Resp	0.1707	0.0556	1	0.1639	0.6
Musculo	0.2314	0.0875	0.3158	1	0.4		Musculo	0.1524	0.1111	0.1639	1	0.4
	HTN	DM	Resp	Musculo	0.2			HTN	DM	Resp	Musculo	0.2

## Discussion

The present study found a high burden of multimorbidity among older adults in six selected districts of Odisha. Overall, 63.2% of participants had two or more chronic conditions. The prevalence varied from 54.2% in Khordha to 74.8% in Cuttack, showing that multimorbidity was not uniformly distributed across the selected districts. These findings are higher than national estimates from LASI-based studies, which have reported lower proportions of older adults with multimorbidity in India [9,19]. In the adjusted model, older adults from Cuttack (aOR = 2.14), Sundargarh (aOR = 2.39), and Balasore (aOR = 1.83) had higher odds of multimorbidity than those from Khordha. These findings suggest that district-level differences may be important when planning geriatric chronic disease services.

The spatial analysis further supported this regional variation. The Global Moran’s I value of −0.5082 indicated negative spatial autocorrelation, suggesting spatial heterogeneity or contrast rather than uniform clustering. Cuttack and Sundargarh appeared as high-burden regional outliers. Similar studies have emphasized the value of examining geographic and regional variation in multimorbidity and older-adult health outcomes [20-24]. However, these findings should be interpreted cautiously because the study was cross-sectional and was conducted in selected districts. The spatial pattern observed in this study should therefore be viewed as an exploratory finding that may guide future district-level research and service planning.

The Jaccard similarity analysis provided additional insight into disease co-occurrence patterns. In Khordha, respiratory and musculoskeletal conditions showed greater overlap (J = 0.3158) than in Sundargarh (J = 0.1639). Hypertension and diabetes showed relatively consistent co-occurrence across Khordha (J = 0.1858) and Sundargarh (J = 0.1983). Jaccard similarity is useful for examining overlap in binary presence-absence data [18], while previous multimorbidity studies have shown that chronic diseases often occur in clinically meaningful clusters among older adults [25,26]. These findings suggest that multimorbidity is not only associated with the number of chronic conditions but also with specific combinations of diseases. Such disease-pair patterns may help inform integrated follow-up services, medication review, physiotherapy support, and chronic disease management [24,27].

Living arrangement was also associated with multimorbidity. Older adults living with children only had higher odds of multimorbidity than those living with both a spouse and children (aOR = 1.75). This finding should be interpreted carefully, as the study did not directly measure caregiving quality, dependency level, or social support mechanisms. Therefore, living arrangements should be considered a possible marker of social vulnerability rather than evidence of a direct causal relationship. Previous studies have shown that spousal loss, intergenerational support, and social capital may influence older adults’ well-being, but these pathways require further investigation in the present context [28,29].

Medication burden was strongly associated with multimorbidity. Participants taking multiple medications had higher odds of multimorbidity than those taking a single medication or no medication (aOR = 1.81). This finding is expected because older adults with multiple chronic conditions are more likely to require multiple medications. However, it also highlights the practical need for regular medication review, counseling, and monitoring for possible drug interactions or inappropriate prescribing. Polypharmacy among older adults has been associated with adverse drug events, drug interactions, treatment complexity, and higher healthcare burden [27].

**Figure 4:**
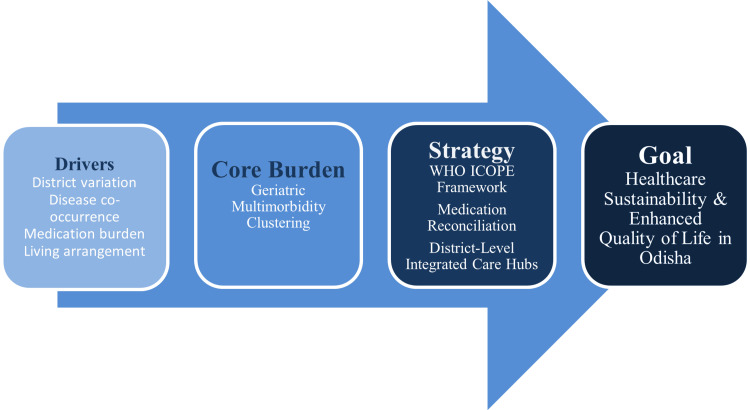
Proposed conceptual framework for district-oriented integrated geriatric multimorbidity care in Odisha. The framework summarizes how district-level variation, disease co-occurrence patterns, medication burden, and living arrangements may inform locally responsive geriatric care planning. The proposed approach emphasizes integrated chronic disease management, medication review, referral linkage, family-based counseling, and alignment with the WHO ICOPE (World Health Organization Integrated Care for Older People) framework. This framework is conceptual and was not tested as an intervention model in the present study.

Overall, the findings support the need for district-oriented geriatric care planning in Odisha. Integrated care hubs that respond to local disease patterns may help improve chronic disease follow-up, medication reconciliation, referral linkage, and family-based counseling (Figure 4). Such a model should be considered a proposed conceptual framework based on the present findings, not a validated intervention model. The WHO Integrated Care for Older People framework supports person-centered assessment and community-level interventions for older adults, and global experiences with ICOPE provide useful lessons for developing integrated geriatric care pathways [24,30]. 

This study has some limitations. Its cross-sectional design limits causal interpretation, and chronic conditions were based on self-reported physician diagnosis, which may involve recall bias or underdiagnosis. The chronic disease profile was based on a limited number of conditions and was not verified through clinical measurements or medical records. As the study was conducted in selected districts and urban wards with higher geriatric concentration, the findings may not fully represent all rural, tribal, or remote populations of Odisha. Future longitudinal studies using broader disease lists, clinical records, and biomarker data may provide stronger evidence on multimorbidity patterns among older adults. 

## Conclusions

This study demonstrates a high burden of multimorbidity among older adults in six selected districts of Odisha, with clear geographic variation and patterns of disease co-occurrence. The negative Global Moran’s I value indicates spatial heterogeneity, suggesting that multimorbidity was unevenly distributed across the selected districts rather than uniformly clustered. The observed differences in disease-pair patterns further suggest that older adults across districts may have distinct care needs. These findings highlight the importance of district-oriented geriatric care planning, regular medication review, and integrated, person-centered chronic disease management. Locally responsive care models aligned with the WHO ICOPE framework may help improve the sustainability and effectiveness of elderly healthcare services in Odisha.
